# Coexistence of Multiple Types of Synaptic Plasticity in Individual Hippocampal CA1 Pyramidal Neurons

**DOI:** 10.3389/fnsyn.2017.00007

**Published:** 2017-03-14

**Authors:** Elke Edelmann, Efrain Cepeda-Prado, Volkmar Leßmann

**Affiliations:** ^1^Institute of Physiology, Otto-von-Guericke UniversityMagdeburg, Germany; ^2^Center for Behavioral Brain Sciences, Otto-von-Guericke UniversityMagdeburg, Germany

**Keywords:** spike timing-dependent plasticity, repeat number, excitatory neurons, BDNF, dopamine, hippocampus, synaptic plasticity, synapse specific LTP

## Abstract

Understanding learning and memory mechanisms is an important goal in neuroscience. To gain insights into the underlying cellular mechanisms for memory formation, synaptic plasticity processes are studied with various techniques in different brain regions. A valid model to scrutinize different ways to enhance or decrease synaptic transmission is recording of long-term potentiation (LTP) or long-term depression (LTD). At the single cell level, spike timing-dependent plasticity (STDP) protocols have emerged as a powerful tool to investigate synaptic plasticity with stimulation paradigms that also likely occur during memory formation *in vivo*. Such kind of plasticity can be induced by different STDP paradigms with multiple repeat numbers and stimulation patterns. They subsequently recruit or activate different molecular pathways and neuromodulators for induction and expression of STDP. Dopamine (DA) and brain-derived neurotrophic factor (BDNF) have been recently shown to be important modulators for hippocampal STDP at Schaffer collateral (SC)-CA1 synapses and are activated exclusively by distinguishable STDP paradigms. Distinct types of parallel synaptic plasticity in a given neuron depend on specific subcellular molecular prerequisites. Since the basal and apical dendrites of CA1 pyramidal neurons are known to be heterogeneous, and distance-dependent dendritic gradients for specific receptors and ion channels are described, the dendrites might provide domain specific locations for multiple types of synaptic plasticity in the same neuron. In addition to the distinct signaling and expression mechanisms of various types of LTP and LTD, activation of these different types of plasticity might depend on background brain activity states. In this article, we will discuss some ideas why multiple forms of synaptic plasticity can simultaneously and independently coexist and can contribute so effectively to increasing the efficacy of memory storage and processing capacity of the brain. We hypothesize that resolving the subcellular location of t-LTP and t-LTD mechanisms that are regulated by distinct neuromodulator systems will be essential to reach a more cohesive understanding of synaptic plasticity in memory formation.

## Introduction

**Figure 1:**
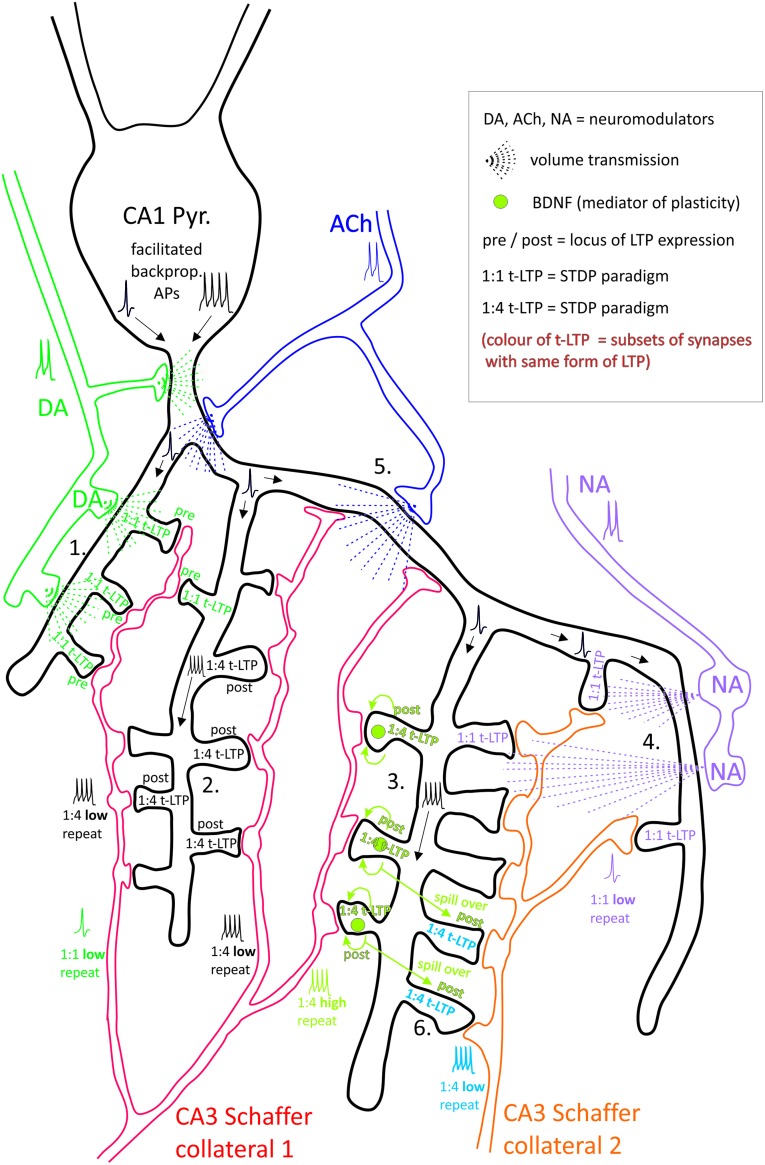
**CA1 pyramidal neuron with (branched) axonal projections (Schaffer collaterals, SC) from two distinct presynaptic glutamatergic CA3 neurons.** Hypothesis: dendritic location of synapses, activity of neuromodulatory input at time point of long-term potentiation (LTP) induction at a specific synapse, and stimulation paradigm determine efficacy of t-LTP at this synaptic site. Synaptic transmission between each presynaptic CA3 neuron and the CA1 neuron takes place potentially at 7 (SC2) and 16 (SC1) ultrastructural synapses, each comprising one presynaptic bouton and one postsynaptic spine. Considered spike timing-dependent plasticity (STDP) paradigms consist of 1:1 or 1:4 pairing of pre- and postsynaptic action potentials (APs) with either high ( >35) or low (<15) number of repeats. Depending on STDP paradigm distinct subsets of ultrastructural synapses undergo pre- or postsynaptically expressed t-LTP (indicated by distinct text colors in the spines; empty spines represent non-potentiated synapses). The different t-LTP types can either be facilitated, enhanced, or inhibited, respectively, by volume transmission of the neuromodulators dopamine (DA), noradrenaline (NA) and acetylcholine (ACh), which can mutually enhance or counteract their effects. In addition, local brain-derived neurotrophic factor (BDNF) release mediates t-LTP specifically in response to short bursts of postsynaptic APs. Specific examples shown: **1.** DA facilitated 1:1 t-LTP (presynaptic expression). **2.** Non-modulated 1:4 low repeat t-LTP (postsynaptic expression). **3.** BDNF-mediated 1:4 high repeat t-LTP (postsynaptic expression). **4.** NA-gated 1:1 t-LTP. **5.** 1:1 t-LTP inhibited by ACh. **6.** Associative t-LTP in response to 1:4 low repeat paradigm at SC 2 by locally restricted BDNF spillover from 1:4 high repeat co-stimulated at SC 1 input. Proximal CA1 neuron dendrite: backpropagation of APs is regulated by DA and ACh. Thus, depending on repeat numbers of STDP paradigms (representing high and low activity states of the brain), activated glutamatergic input, and STDP paradigm distinct ultrastructural synapses are fine-tuned in plasticity.

The discovery of the synapse as a connection between two sets of neurons in the early 20th century marked a new era of neuroscience. The synapse was identified as the most suitable structure for memory storage and for controlling the flow of information from one neuron or brain area to another. To date, it is known that persistent synaptic activation induces bidirectional plasticity leading to either strengthening or weakening of the connections between synaptically connected neurons, commonly called long-term potentiation (LTP; Bliss and Lomo, 1973; Hölscher, 1999; Malenka and Nicoll, 1999) or long term depression (LTD; Lynch et al., 1977; Collingridge et al., 2010), respectively. These kinds of long-lasting changes of synaptic transmission are accepted cellular models for learning and memory and need to be studied to understand the biochemical processes underlying synaptic plasticity under physiological and pathophysiological conditions. Associative forms of synaptic plasticity induced by repeated or persistent activation of both connected neurons were postulated by Hebb (1949). These LTP and LTD phenomena can be induced by different stimulation types and in different brain circuits analyzed in several animal species and at varying age. When considering the effects of neuromodulators and mediators of synaptic plasticity the multitude of LTP and LTD paradigms inevitably results in a complex pattern of neuromodulation (for brain-derived neurotrophic factor (BDNF): reviewed in Gottmann et al., 2009; Edelmann et al., 2014; for dopamine (DA): reviewed in Pawlak et al., 2010; Edelmann and Lessmann, 2013). Depending on the strength and duration of LTP or LTD induction, some of the generated results might be difficult to interpret in terms of models for physiological relevant processes involved in learning and memory. For example, very long-lasting and strong stimuli commonly used to establish LTP/LTD might not properly reflect realistic patterns of neuronal activity that can be observed in a behaving animal, *in vivo*. Furthermore, many LTP/LTD results are obtained from recordings that average synaptic responses of groups of neurons rather than looking at the level of single cells. While responses from groups of neurons might provide a better insight into synaptic changes at the network level, recordings at the single cell level allow investigating synaptic function with sufficient spatial resolution to disentangle subcellular and molecular differences of synaptic plasticity in the same neuron. We hypothesize that the location of a synaptic input onto a postsynaptic neuron along its dendritic tree decides about the direction (i.e., LTP or LTD), the magnitude, and the expression mechanism of the synaptic modification that is induced. This decision is regulated by the local neuromodulatory microenvironment (including DA, noradrenaline (NA), acetylcholine (ACh) and BDNF) in the vicinity of the synaptically activated dendritic location (compare Figure 1).

When searching for electrical processes that contribute to dendritic location specific synaptic plasticity, backpropagating action potentials (bAP) come into play. Active backpropagation of sodium-dependent action potential (AP) into dendrites (see e.g., Stuart and Sakmann, 1994; Stuart et al., 1997), provides an ideal associative signal to the dendrites for Hebbian synaptic plasticity (Magee and Johnston, 1997). This feature is essential for a type of synaptic modification, called spike timing-dependent plasticity (STDP). STDP can be induced by exactly timed repetitive activations of either single or multiple spikes in pre- and postsynaptic neurons and was shown for many synapses in different brain regions (reviewed in e.g., Dan and Poo, 2006; Caporale and Dan, 2008; Debanne and Poo, 2010; Feldman, 2012; Markram et al., 2012). STDP was also described for mossy fiber-CA3 and Schaffer collateral (SC)-CA1 synapses in the hippocampus. Pairing with the sequence, presynaptic AP first and postsynaptic spike a few ms thereafter, usually leads to potentiation (timing (t)-LTP), while the opposite sequence (i.e., post-pre pairing) leads to depression of synaptic transmission resulting in t-LTD (but see Debanne et al., 1994, 1997; Fino et al., 2005; Letzkus et al., 2006; Ruan et al., 2014, for anti-hebbian synaptic plasticity). However, as is also true for STDP in other brain areas, results at SC-CA1 synapses from different studies often do not match very well. This is most likely due to specific differences in experimental conditions that result in subtle but important changes in postsynaptic Ca^2+^dynamics (reviewed in Buchanan and Mellor, 2010). Moreover, secreted neuromodulators such as DA or NA (reviewed in Pawlak et al., 2010; Edelmann and Lessmann, 2013; Fremaux and Gerstner, 2015) shape the type and magnitude of STDP. Last but not least, synaptically released mediators of plasticity such as BDNF crucially regulate the efficacy of STDP (e.g., Sivakumaran et al., 2009; Lu et al., 2014; Edelmann et al., 2015).

In this article, we will focus on the coexistence of different forms of STDP at hippocampal SC-CA1 synapses and their distinctly different modulation by BDNF and DA, and by their respective receptors. We believe that STDP is a valuable tool/model to study cellular processes which might be involved in learning and memory in the hippocampus (but see Lisman and Spruston, 2010), and will focus our discussion on stimulation scenarios which allow investigating coexisting types of plasticity that can be induced at the same synapses by more or less subtle changes in STDP paradigms.

## STDP as a Model to Encode Memory Engrams that Are Activated and Recruited by Different Levels of Neuronal Activity

Timing (t)-LTP or t-LTD that are induced by STDP protocols have been observed in response to various protocols and in different circuits and brain areas (summarized in e.g., Dan and Poo, 2006; Caporale and Dan, 2008; Markram et al., 2011; Feldman, 2012). So called canonical forms of STDP are induced by pairing one presynaptic with one postsynaptic bAP (e.g., Bi and Poo, 1998). These seminal experiments were performed in cultured hippocampal neurons developing in the absence of modulatory (i.e., dopaminergic, cholinergic, serotonergic) inputs. Later on, canonical STDP was shown also for SC-CA1 synapses in acutely isolated hippocampal slices. Nevertheless, successful protocols and signaling mechanisms underlying t-LTP and t-LTD are partially divergent between studies (compare Buchanan and Mellor, 2010) and dependent on experimental details (e.g., Edelmann and Lessmann, 2011, 2013). Using a single experimental approach but different STDP protocols, we recently showed that two types of spike timing-dependent LTP can coexist at the same hippocampal SC-CA1 synapses (Edelmann et al., 2015). These two types of t-LTP were induced either by a canonical STDP paradigm (1:1, 70–100 repeats (x) at 2 s intervals) or when presynaptic activation was combined with a postsynaptic burst of four APs (1:4 protocol, 25–30 repeats (x), 2 s intervals). The results of these experiments demonstrated similar net potentiation in both paradigms, similar levels of t-LTD with change of stimulation sequence and similar dependency on NMDA receptor (NMDAR) activation. Strikingly, when we determined the cellular mechanisms (including pre- vs. postsynaptic expression) engaged to mediate the synaptic potentiation and the role that different neuromodulators play in enabling t-LTP, both protocols were clearly different (see “Input Specific Involvement of Neuromodulators Allows Coexistence of Multiple forms of STDP” Section). Similarly, coexistence of distinguishable types of LTP has been described previously for LTP relying on tetanic or theta burst synaptic stimulation. Recently, Wang et al. (2016) showed that NMDAR dependent and metabotropic glutamate receptor (mGluR) dependent LTP can coexist at SC-CA1 synapses. Furthermore, Urban and Barrionuevo (1996) described coexisting forms of hebbian and non-hebbian LTP at hippocampal mossy fiber synapses. Both of these two previously mentioned data sets suggest that two memory engrams can be stored with different mechanisms at a distinct set of synapses that connect a presynaptic with a postsynaptic neuron. These coexisting forms of LTP could result from differentially recruited LTP mechanisms that are switched on either by high or by low brain activity patterns that can be observed during memory formation (Figure 1). In accordance with these studies on conventional LTP, the 1:4 STDP paradigm (compare above) used to induce t-LTP at SC-CA1 synapses would rely on signaling pathways and mechanisms that are activated during high brain activity, while the canonical 1:1 t-LTP protocol mechanisms would be ideally suited to be engaged for computing synaptic plasticity during low brain activity states.

A similar coexistence of plasticity mechanisms as described in the last paragraph for LTP is also known for LTD. Also here, different types of LTD can coexist that are independently activated by either NMDAR or mGluR activation during LTD induction (Nicoll et al., 1998). However, because more research is focused on t-LTP rather than on t-LTD, a similar coexistence of different types of t-LTD induced by STDP protocols has—to our knowledge—not yet been described. Functionally, it remains to be determined whether plasticity mechanisms recruited in response to burst paradigms for t-LTP (and t-LTD) mimic high activity states (i.e., gamma frequency oscillations) of the brain during wakefulness, while canonical STDP protocols induced pathways are involved in consolidation of memory during resting periods which are dominated by slow wave EEG activity in the theta and delta frequency range. In this respect, future experiments testing whether STDP paradigms elicited synchronously with sharp wave ripple oscillations (that occur during memory replay in CA1 *in vivo*, and are also observed in acute slices *in vitro*; compare Draguhn et al., 2000; Buzsáki, 2005) affect t-LTP expression, could tell whether physiologically relevant brain activity states can regulate this type of plasticity also *in vivo*.

Taken together these observations highlight the coexistence of different types of synaptic plasticity at distinct subsets of synapses onto a postsynaptic neuron in a given synaptic circuit. Further, it is tempting to speculate that these distinguishable mechanisms of plasticity enable encoding and retrieval of memory during different levels of brain activity. We hypothesize that different subsets of synapses even of the same neuron might contribute via t-LTP to memory formation during both activity states. This could be tested in t-LTP experiments that identify potentiated synapses e.g., by spine Ca^2+^ imaging. In case distinct spines of the same neuron contribute to t-LTP that was induced in synchrony with gamma rather than theta oscillations, they should be identified with such an approach.

## Input Specific Involvement of Neuromodulators Allows Coexistence of Multiple Forms of STDP

**Figure 2:**
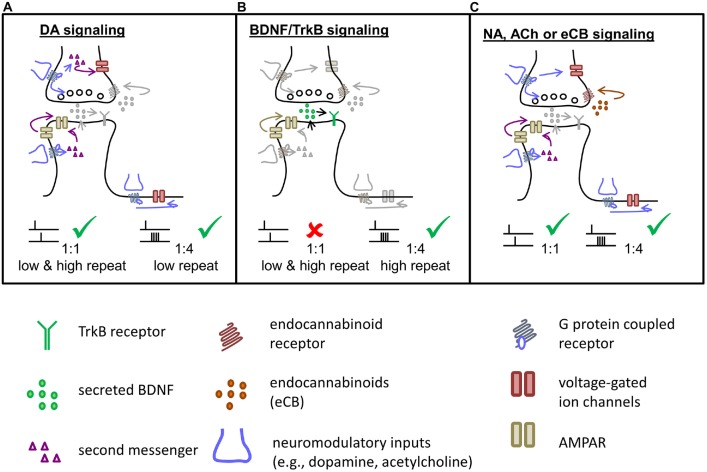
**Signaling cascades involved in t-LTP induce by different STDP paradigms at distinct chemical synapses.** Specific STDP protocols activate different signaling and expression mechanisms in pre- and postsynaptic parts of the synapse to induce specific types of synaptic plasticity. **(A)** Putative dopaminergic (DA) signaling mechanisms underlying t-LTP induced either with a canonical 1:1 paradigm or with a burst paradigm at low repeat number. Pre-, post- and extrasynaptic mechanisms might be involved. **(B)** BDNF/TrkB signaling involved in STDP induced by burst protocols. t-LTP induction with a 1:4 protocol recruits autocrine postsynaptic mechanisms by BDNF and TrkB signaling. **(C)** NA, ACh or endocannabinoid (eCB) signaling can contribute by pre- and postsynaptic mechanisms similar to DA, in regulating the efficacy of t-LTP at distinct and/or overlapping ultrastructural synapses. Lower panel: description of symbols.

An important role for neuromodulation in regulating synaptic plasticity was reported for different types of synaptic plasticity and was observed in different brain regions. In this respect, a large body of evidence stresses the important role of ACh, DA, intracellular cAMP elevation and BDNF signaling, respectively, in gating, facilitating or even mediating signaling events leading to synaptic plasticity (see e.g., cAMP: Otmakhova et al., 2000; DA/cAMP: Navakkode et al., 2010; Sheynikhovich et al., 2013; Otani et al., 2015; ACh: Nakauchi and Sumikawa, 2012; BDNF: Sivakumaran et al., 2009; Schjetnan and Escobar, 2012; Schildt et al., 2013, compare Figure 2). These same neuromodulators were also reported to be essential for establishing STDP (see e.g., ACh: Couey et al., 2007; Goriounova and Mansvelder, 2012; NA/ACh: Seol et al., 2007; DA: Pawlak and Kerr, 2008; Zhang et al., 2009; Edelmann and Lessmann, 2011, 2013; Cassenaer and Laurent, 2012; Yang and Dani, 2014; endocannabinoids (eCB): Cui et al., 2015, 2016; BDNF: Edelmann et al., 2015; Monoamines: He et al., 2015; compare Figure 1).

For hippocampal STDP, we recently described two distinguishable forms of t-LTP that depend on the availability of different neuromodulators/neuromediators. The canonical STDP paradigm induced by 1:1 pairing at SC-CA1 synapses (1:1, 70–100 repeats, 0.5 Hz) is dependent on DA signaling via D1 receptor activation (Edelmann and Lessmann, 2011), while the 1:4 protocol (1:4, 25–30 repeats, 0.5 Hz) recruits postsynaptic BDNF and postsynaptic TrkB receptor activation, respectively, to allow successful induction/expression of t-LTP (Edelmann et al., 2015). Notably, the endogenous DA that is involved in neuromodulation is not released from the pre- or postsynaptic structures that undergo the recorded potentiation. Rather, it is likely released from axon terminals of dopaminergic neurons projecting somewhere nearby the potentiated synapses (Figure 1). In contrast, BDNF is released from the same subset of glutamatergic synapses that are subjected to potentiation during t-LTP and it is released directly in response to AP firing from the postsynaptic site. This is the reason why DA, NA, ACh and related transmitters are considered as pure neuromodulators, while in contrast BDNF is considered as a mediator of synaptic plasticity (see above) albeit additional neuromodulation at neighboring synapses/neurons is possible.

**Figure 3:**
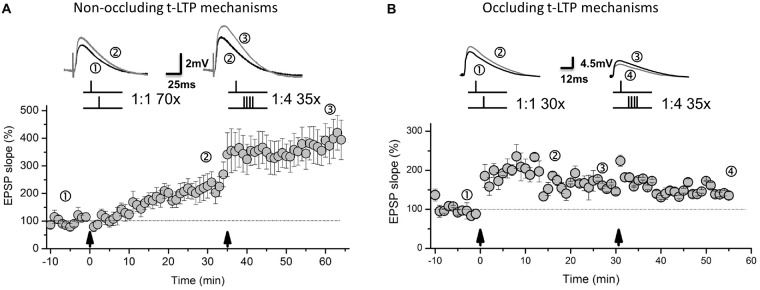
**Distinct types of t-LTP can either occlude one another or can be non-occluding at SC-CA1 synapses. (A)** Depending on STDP induction paradigm, non-occluding t-LTP types can be induced subsequently in the same CA1 pyramidal neuron by a canonical 1:1 paradigm (70 repeats, at 0.5 Hz) and subsequent burst 1:4 stimulation (35 repeats, at 0.5 Hz). **(B)** However, small changes in the number of pairings for the 1:1 protocol (30 instead of 70 repeats, at 0.5 Hz) lead to occluding t-LTP types. Pairing frequency and stimulus pattern remained unchanged in the different approaches to induce non-occluding and occluding types of t-LTP. Original traces represent representative EPSP traces during different phases of the experiments (see numbers). Panel **(A)** is modified from Edelmann et al. (2015). Panel **(B)** unpublished own observations. For overall experimental design, see Edelmann et al. (2015).

Interestingly, both types of t-LTP (1:1 = DA-regulated; 1:4 = BDNF-mediated) can be induced independently and subsequently at a given subset of SC-CA1 synapses (Figure 3, for methods see Edelmann et al., 2015). Whether the released DA gives rise to altered BDNF secretion or whether secreted BDNF facilitates DA release, thereby allowing direct crosstalk between these two neuromodulatory systems remains to be investigated (compare Li et al., 2011; Navakkode et al., 2012). Nevertheless, the 1:1/DA t-LTP remains unaffected if BDNF signaling is inhibited and—vice versa—the 1:4/BDNF t-LTP remains unaltered if DA signaling is blocked (see Edelmann and Lessmann, 2011; Edelmann et al., 2015). Interestingly, variations in repeat numbers or STDP stimulation pattern employed to induce t-LTP can alter the requirements for DA and/or BDNF neuromodulation and can also change the independence of the two distinct types of t-LTP at SC-CA1 synapses. In this respect, we observed that STDP induced by a 1:1 paradigm with only 30 repeats at 0.5 Hz occludes t-LTP induced by the 1:4 protocol with 35 repeats, pointing to a loss of independent synaptic potentiation by a change just in the repeat number of the 1:1 STDP protocol (0.5 Hz; Edelmann et al., unpublished data, Figure 3).

Neuromodulators can affect—among other things—neuronal excitability (AP firing and propagation properties), transmitter release, morphology (dendritic branching, spine shape) and molecular outfit of synaptic structures. Importantly, neuromodulatory transmitters like ACh, DA and NA can shape the backpropagation of APs or calcium spikes in the dendrites by different G-protein induced actions (see e.g., Hoffman and Johnston, 1999; Frick and Johnston, 2005; Sweatt, 2016) and thereby “prime” certain locations along the postsynaptic dendrite (e.g., distal or proximal dendrites) for synaptic plasticity.

Moreover, the microarchitecture of a glutamatergic synapse that is susceptible to undergo LTP might play an important role. For example, the density and proximity of axon terminals from different neuromodulator secreting cells (ACh, DA, NA), as well as firing mode and firing rates of these cells can enhance or multiply the versatility of neuromodulatory mechanisms for memory encoding (compare Figure 1). Future investigations addressing the spatial and temporal resolution of this neuromodulator signaling will be of utmost importance to delineate their combined role in synaptic plasticity. Understanding these principles in *in vitro* preparations might serve as a basis for subsequent *in vivo* analysis. However a suitable experimental *in vivo* approach remains to be identified for those investigations.

## Coexistence of STDP by Engaging Different Mechanisms of LTP Expression

Protein synthesis independent of early LTP can be either expressed presynaptically by increased neurotransmitter release, postsynaptically by phosphorylation and lateral translocation of existing AMPA receptors, or by incorporation of stored AMPA receptor containing vesicles into the postsynaptic membrane. For the two major glutamatergic pathways in the hippocampus (SC-CA1 and MF-CA3) the main locus of expression of early LTP diverges. Postsynaptic expression seems to be the most plausible mechanism for early LTP at SC-CA1 synapses, while at MF-CA3 synapses, presynaptic expression takes place (for SC LTP: Lu et al., 2014, for MF LTP: Nicoll and Schmitz, 2005). In contrast, coexistence of two forms of LTP with different pre- and postsynaptic expression mechanisms has been shown for thalamocortical synapses in the anterior cingulate cortex (Li et al., 2010; Koga et al., 2015). Both forms of LTP are suggested to act in concert for mediating pain and anxiety states (Koga et al., 2015). The presynaptic expression mechanism was reported to involve adenylate cyclase (AC) and protein kinase A (PKA) signaling, while the postsynaptic LTP mechanism recruits protein kinase M zeta (PKMζ; Li et al., 2010; Koga et al., 2015). A similar coexistence of different LTP expression mechanisms at the same synapses was also shown for the thalamic input to the lateral nucleus of the amygdala (Shin et al., 2010). Shin et al. (2010) demonstrated either postsynaptically expressed LTP that was induced by a pairing protocol or presynaptically induced and expressed LTP by purely presynaptic low frequency stimulation. As a possible function, the authors suggested, that varying activity patterns of neurons in behaving animals might produce distinct levels of postsynaptic depolarization that recruit different types of synaptic plasticity to encode and retrieve conditioned fear memories (Shin et al., 2010). While they showed that cannabinoids might be involved in the postsynaptically expressed LTP, further details on the presynaptic mechanism were not reported. However, in a related preparation the increase of neurotransmitter release in presynaptic LTP was shown to be mediated by cAMP/PKA signaling and involving the active zone protein RIM1α (Castillo et al., 2002; Chevaleyre et al., 2007; Shin et al., 2010).

For t-LTP induced by STDP protocols at hippocampal SC-CA1 synapses a postsynaptic locus of expression had been generally assumed—but it was not investigated until recently. In this respect, we could show lately that at SC-CA1 synapses different forms of t-LTP can coexist independently (Edelmann et al., 2015). While the canonical LTP protocol induced by a 1:1 stimulation paradigm was expressed in the absence of postsynaptic AMPA receptor incorporation or phosphorylation, as indicated by unaltered AMPA/NMDA ratio following t-LTP, we found a significant reduction of paired pulse facilitation after successful induction of this 1:1 t-LTP. For the 1:4 burst t-LTP, we found a clear postsynaptic expression by incorporation of GluA1 containing AMPA receptors into the postsynaptic membrane. Both types of pre- or postsynaptically expressed t-LTP can be activated independently and do not occlude one another (Edelmann et al., 2015, compare Figure 3). In a similar fashion, additional yet to identify signaling cascades for t-LTP expression might be recruited by distinct synaptic stimulation paradigms, likely giving rise to a multitude of different forms of STDP. These types might coexist in a given cell, and share the same subset of synapses, while recruiting different pre- and postsynaptic loci to mediate the synaptic changes (i.e., pre- vs., postsynaptic) and while employing varying molecular mechanisms of expression of synaptic plasticity.

The discussed *in vitro* experiments are crucial for defining the toolbox of neuromodulation-dependent plasticity mechanisms that can shape LTP within a neuron’s dendritic tree. However, experimental approaches that would enable to investigate this synapse specific modulation with the same spatial resolution in CA1 *in vivo* remain to be established.

## Location-Dependent STDP in Hippocampal Schaffer Collateral—CA1 Synapses: Contribution of Basal and Apical Dendrites of CA1 Neurons, Presynaptic Terminals and Deep and Superficial CA1 Neurons

### LTP in Apical vs. Basal Dendrites in CA1

If our hypothesis is correct that discriminable dendritic compartments of a neuron undergo LTP in response to distinctly different induction paradigms, and that this is at least in part due to the divergent neuromodulatory microenvironment, it should be possible to assign specific types of LTP to specific locations along the dendrite. The apical (i.e., oblique) and basal dendrites of CA1 pyramidal neurons are the main target regions of the presynaptic SCs in the CA1 area (Megías et al., 2001; Witter, 2007). While distantly located neurons in the CA3 region project primarily to the apical dendrites of CA1 neurons, nearby CA3 neurons project more heavily to the basal dendrites (Spruston, 2008). Due to this circuit specialty, different CA3 neurons can—depending on dendritic location—encode specific information at distinct synapses of a given CA1 neuron (Witter, 2007; Li et al., 2016; compare Figure 1). Such location specific differences between synaptically encoded information at distinct synaptic locations of CA1 neurons were recently shown by Mahmmoud et al. (2015) in a hippocampus-dependent learning task. They observed an increase in spines (mushroom types) after training in a radial arm maze. However, this effect was specific only for apical and not for basal dendrites of CA1 pyramidal neurons. Since the CA1 region seems to be involved in this learning task, the data suggested that only CA3 inputs to apical dendrites (distant connections) were activated in this learning task.

Similarly, divergent types of synaptic plasticity expressed at basal and apical dendrites were demonstrated also in other reports. For example, DA-dependent LTP was shown on the one hand to be induced at both, basal and apical dendrites of CA1 neurons. On the other hand, the underlying signaling mechanisms were distinctly different between both synaptic locations. Thus, while the DA-dependent LTP at basal dendrites was shown to depend on the activation of L-type voltage gated calcium channels and NMDARs, DA-dependent LTP at apical dendrites only recruited NMDAR receptors (Navakkode et al., 2012). Furthermore, the authors showed that the apical DA-dependent LTP was induced and maintained only in the presence of BDNF. Recently, it was also shown that the magnitude of LTP *in vivo* is larger in basal than in apical dendrite synapses of CA1 pyramidal neurons. Region-specific application of low doses of the D4 type DA receptor agonist PD168077 attenuated LTP in basal but not in apical CA1 dendrites (Li et al., 2016). In contrast, cholinergic modulation seems to be more important for the neuromodulation of LTP at specific sites of apical dendrites in the CA1 area (Leung and Peloquin, 2010; Li et al., 2016). Together these observations are consistent with a dendrite location specific modulation of synaptic plasticity in CA1 pyramidal neurons.

Using systemic and cellular approaches, several reports suggested that a spatial gradient of LTP relevant receptors and channels along CA1 dendrites might be the basis for the observed location-dependent synaptic plasticity mechanisms that also affect hippocampus dependent learning. It was suggested that L-type voltage gated calcium channels are expressed preferentially in the soma, basal dendrites, and in proximal—but not distal—apical dendritic regions (Tippens et al., 2008; Navakkode et al., 2012). For AMPARs and hyperpolarization-activated cation conductances (Ih), a somato-dendritic gradient was described for apical dendrites of CA1 neurons (Magee, 1998; Andrasfalvy and Magee, 2001; Lörincz et al., 2002; Nicholson et al., 2006). It remains to be investigated how this differential distribution of these channels might relate to the differences in LTP magnitude and D4 receptor dependence of LTP between apical and basal CA1 dendrites. Overall these results strongly support the concept that the neuromodulatory microenvironment in fact determines the location of potentiated synapses in CA1 pyramidal neuron dendrites.

### Dopamine, NMDA and AMPA Receptor Distribution

DA is one important and possibly representative neuromodulator shaping synaptic plasticity in the hippocampus. The CA1 region receives inputs from the ventral tegmental area or locus coeruleus (Gasbarri et al., 1994; Smith and Greene, 2012). These dopaminergic fibers show a soma distance-dependent gradient in the innervation density of the CA1-region along apical and basal dendrites. This gradient in the distribution of dopaminergic fibers is paralleled by an uneven subcellular localization of DA receptor subtypes and thus also altered affinity towards DA along the dendrites (Swanson et al., 1987; Goldsmith and Joyce, 1994; Yao et al., 2008; Smith and Greene, 2012; Rosen et al., 2015; Li et al., 2016). In terms of DA-dependent regulation of t-LTP, this distribution of dopaminergic fibers and receptors in the CA1 area could enable manifestation of t-LTP at discernable synapses of the same neuron in response to distinct STDP paradigms (e.g., 1:1 vs. 1:4) which might recruit DA signaling via distinct receptor subtypes (Cepeda-Prado et al., unpublished data). We hypothesize that such distinguishable forms of DA-dependent t-LTP might be encoded in different stretches of the apical, oblique or basal dendrites of CA1 neurons. Moreover, this hypothesis suggests that these t-LTP forms might exist independently and parallel within the same neuron. This interpretation is supported by the finding that in CA1, D1 receptors are expressed preferentially in the apical dendritic spines, while D5 receptors are expressed in the shaft region (between spines) of CA1 pyramids where they form synaptic contacts with GABAergic interneurons (Yao et al., 2008). Synaptic plasticity regulated by D4 receptor subtypes is most prominent for basal dendritic sites (Li et al., 2016). Future experiments aiming to activate release of endogenous DA (e.g., by optogenetic methods) in confined dendritic branches of CA1 pyramidal neurons could help to shed light on these synaptic site specific DA actions.

As mentioned before a variety of different studies showed a synaptic subtype specific and region-specific variability in synaptic AMPA and NMDAR expression. All synapses (perforated and non-perforated synapses) contain NMDARs, while perforated synapses (i.e., the majority of synapses, harboring two or more delimited postsynaptic densities in the same spine) show additionally a distance-dependent increase in the expression level of AMPARs from proximal to distal regions (Andrasfalvy and Magee, 2001; Nicholson et al., 2006; Nicholson and Geinisman, 2009). However, 40% of all non-perforated synapses lack any expression of AMPARs and are thought to represent a reserve pool of silent synapses for nascent functional connections (e.g., Nicholson et al., 2006; Toni et al., 2007; Nicholson and Geinisman, 2009). Thus, parallel and independent synaptic plasticity types induced by different STDP paradigms might recruit and strengthen existing AMPAR containing synapses, or can activate and use nascent synapses by unsilencing NMDAR only synapses.

Thus, the spine type (i.e., perforated vs. non-perforated) sets the stage for the mechanism of LTP expression that can take place at this spine, thus representing another cellular process that decides which type of plasticity can be brought about at this very location.

### BDNF and TrkB Receptor Distribution

A synapse specific microenvironment for t-LTP expression is also likely to be created by the differential availability of BDNF/TrkB signaling at individual synapses. Postsynaptically mediated BDNF-dependent types of t-LTP are presumably expressed at specific dendritic sites, where BDNF is synthesized or delivered (compare Figure 1). Alternatively (or in addition), BDNF-dependent t-LTP could be restricted by synapse specific dendritic expression of the cognate TrkB receptor.

Importantly, expression of BDNF mRNA is heavily regulated by neuronal activity in the BDNF synthesizing glutamatergic neurons. Furthermore, different BDNF mRNA, splice variants were shown to be transported with varying efficiencies into dendrites. This splice variant dependent translocation—followed by local translation into BDNF protein within the dendrite—gives rise to restricted localization of BDNF in the cell body, and proximal vs. distal dendritic compartments (Baj et al., 2011). Upregulation of individual BDNF splice variants can thus eventually result in a highly selective and spatially restricted TrkB activation, given the local BDNF containing vesicles are released upon synaptic activation (Hartmann et al., 2001; Edelmann et al., 2015). In terms of BDNF-dependent t-LTP, which is mediated postsynaptically by TrkB receptor activation (compare Edelmann et al., 2015), the above mentioned BDNF mRNA species-dependent transport could lead to a spatial segregation of BDNF supply at distinguishable synapses depending on the applied pattern and repeat number of a STDP paradigm. Such segregation could enable restriction of BDNF-dependent t-LTP to BDNF rich dendritic subdomains.

Apart from postsynaptic sites, BDNF can also be secreted from presynaptic neurons (for a recent review, see Edelmann et al., 2014), thus further enhancing the variability of BDNF availability along dendritic synaptic sites. This uneven BDNF distribution is paralleled by a most likely non-uniform dendritic localization of TrkB receptors, resulting in altered BDNF-dependent synaptic plasticity (compare Zakharenko et al., 2001). For example, Drake et al. (1999) showed intense TrkB receptor labeling in axons of CA1 neurons, but also in terminals of CA3 pyramidal neurons. Additionally, TrkB immunoreactivity was also observed in dendritic spines in this study. Together these data suggest that BDNF/TrkB-dependent LTP (including t-LTP) can be restricted to specific subsets of synapses of a neuron and thereby allow pre- and postsynaptic modulation of glutamatergic neurotransmission. The varying locations for this spatially restricted BDNF-mediated LTP possibly depend on the synaptic activity paradigm that is used for LTP induction (compare Edelmann et al., 2014). Last but not least, TrkB receptors are also present in GABAergic, cholinergic and monoaminergic terminals, which might be involved in the recently described transactivation of TrkB receptors (e.g., Huang et al., 2008; Nagappan et al., 2008). Thereby neuromodulatory transmitter systems can even crosstalk to BDNF/TrkB signaling adding an additional level of complexity to the neuromodulator microenvironment for t-LTP induction at a specific synaptic site.

### Dendritic Filtering of Action Potentials and Presynaptic Release Properties

Independent from spatial gradients of receptors and the heterogeneity of apical and basal dendrites (compare above), STDP induction is heavily dependent on successful backpropagation of APs. In this respect it seems reasonable to assume that factors regulating this backpropagation are likely to facilitate synaptic plasticity in more distal parts of the apical and basal dendrites of the CA1 area (compare Figure 1). This is because dendritic filtering which is controlled by regulation of active conductances (i.e., voltage gated Na^+^, K^+^ and Ca^2+^ channels) in dendrites is likely to erase (some of the) bAPs. This has almost certainly more dramatic consequences for weaker (1:1 stimulation) than stronger (1:4) STDP paradigms. Whether repeat number, pairing frequency or pattern of postsynaptic bursts in STDP paradigms is the strongest determining factor remains to be tested. However, it seems plausible that more robust stimulations might be suited to overcome any attenuation of bAPs in dendrites (Golding et al., 2001; Bernard and Johnston, 2003).

Apart from the heterogeneity of postsynaptic structures, also presynaptic inputs are believed to be heterogeneous and can lead subsequently to cellular domain specific t-LTP types. For example, Dobrunz and Stevens (1997) described heterogenous transmitter release probability and different sizes of the readily releasable pool of transmitter vesicles in SC terminals projecting to CA1 pyramidal neurons (Dobrunz and Stevens, 1997; Nusser et al., 1998). In concert with postsynaptic subdomains that are more eligible to potentiation (compare above), the activation of different presynaptic inputs might thus lead to distinct and coexisting types of plasticity.

### Deep vs. Superficial CA1 Pyramidal Neurons

In addition to the pre- and postsynaptic heterogeneity along the somato-dendritic axis, multiple forms of t-LTP and its putative role in memory could be explained by the variability of distinct populations of CA1 neurons with respect to their distinguishable transcriptome profiles. Differences in morphology of superficial (towards Stratum oriens) vs. deep CA1 pyramidal neurons (close to Stratum radiatum) were already described by Lorente de Nó (1934) and could reflect a parallel difference in transcriptome and proteome between these sets of neurons. Deep and superficial neurons are born during distinct neurogenic windows (i.e., neurogenesis in superficial layers is 1–2 days delayed compared to deep layers), are driven by distinct afferent inputs, encode different environmental features, and serve in different forms of learning (Mizuseki et al., 2011; Danielson et al., 2016). Deep pyramidal cells in CA1 are more active and tend to burst compared to superficial pyramidal neurons (Mizuseki et al., 2011). However, it is yet not determined whether t-LTP is differentially gated and expressed in deep and superficial CA1 pyramidal neurons, since most studies assumed homogenous function of CA1 pyramidal neurons.

The heterogeneity of apical and basal dendrites and the described somato-dendritic gradients of receptors, transmitter release probabilities and ion channels might be important for the location specific activation of distinct synapses to allow independent coexistence of multiple types of t-LTP in the same individual neuron. Furthermore recent studies suggest functional differences of deep and superficial CA1 pyramidal neurons, which might underlie different and coexisting mechanisms of synaptic plasticity. Whether the subtle changes in induction paradigms that lead to differential success of t-LTP along the dendritic tree of a CA1 pyramidal neuron *in vitro* are crucial to understand hippocampal memory formation *in vivo*, remains a challenging question to be tackled by future experiments.

## Synaptic Plasticity in Large and Small Spines

According to our central hypothesis of this article, that the synaptic microenvironment shapes the responsiveness of a given synapse to undergo a specific type of t-LTP, spine morphologies need to be taken into account. Dendritic spines are tiny membrane protrusions, consisting of a head (volume ~0.05 μm^3^) anchored to the dendritic shaft by the spine neck (length ~0.5 μm; Harris and Stevens, 1989). They have multiple sizes and shapes and are classified according to their structure as filopodial and thin spines (commonly called small spines), as opposed to stubby, fenestrated and mushroom-shaped spines (also known as large spines; Hering and Sheng, 2001). Moreover, spines are equipped with an electron-dense region, termed postsynaptic density (PSD) composed of hundreds of proteins shaping the structure, stability and function of these postsynaptic protrusions (Kennedy, 1997, 2000; Nimchinsky et al., 2002; Okabe, 2007). Structural analysis and electrophysiological experiments have shown that spine head size, PSD area (Katz et al., 2009), and spine neck electrical resistance, respectively, exhibit an increasing proximo-distally gradient along basal and oblique dendrites (Harnett et al., 2012). Such structural variability confers to the dendritic spines the ability to control postsynaptic calcium ion concentration [Ca^2+^] in a compartmentalized fashion, which is mainly due to the spine neck anatomy (Lisman, 1989; Guthrie et al., 1991; Müller and Connor, 1991; Yuste et al., 2000). Thus, a long slender neck, usually related to small spines, assigns enormous control over the diffusional coupling between spine head and parent dendrite, resulting into a chemical and electrical compartmentalization. In contrast, large spines have broader necks allowing still limited but greater spine-dendrite interaction than small spines (Bloodgood and Sabatini, 2005; Noguchi et al., 2005; Grunditz et al., 2008). Indeed, STDP experiments in which two-photon glutamate uncaging was paired with back-propagating APs revealed that spines dynamically interact with the parent dendrite (e.g., head enlargement together with spine neck shrinking and swelling). These dynamics were shown to rely on cytoskeleton dynamics, protein translocation, PSD reorganization and protein synthesis (Majewska et al., 2000; Araya et al., 2006, 2014; Grunditz et al., 2008; Bosch et al., 2014).

In CA1 pyramidal neurons structural plasticity requires activation of BDNF/TrkB signaling (Tanaka et al., 2008), that together with regenerative local dendritic APs, termed dendritic spikes, have been associated with synaptic plasticity at distal but not proximal synapses along the dendrite (Gordon et al., 2006). Moreover, Matsuzaki et al. (2004) demonstrated that long-lasting enlargement of spine heads positively correlates with increases of AMPAR conductance in a given spine, which was observed specifically in small rather than large spines. In these larger spines, the head enlargement lasted only few minutes before returning to its original size. Accordingly, AMPAR currents remained unaffected in these larger spines (Matsuzaki et al., 2001, 2004). Overall, it seems plausible that the spine architecture in itself serves a modulatory role for synapse specific t-LTP, well before any neuromodulatory transmitter gradients in its vicinity (compare Figure 1) come into play.

Recently, we demonstrated that two different STDP paradigms (1:1, 70 repeats, 0.5 Hz compared to 1:4, 35 repeats, 0.5 Hz) differentially affect AMPAR conductance at the potentiated synaptic sites. Synaptic plasticity induced with the 1:4 rhythm significantly increased the AMPAR mediated currents due to enhanced trafficking of GluA1 subunit containing receptors, whereas for the 1:1 paradigm synaptic AMPAR conductances remained unaffected (Edelmann et al., 2015). We also found that the increase of synaptic efficacy achieved with 1:4, but not by the 1:1 paradigm, was BDNF dependent. Although these two STDP paradigms induce different forms of synaptic plasticity, it remains to be determined whether they can specifically impact distinct spines (or spine types).

Together these data suggest that the morphological and functional changes that spines undergo in response to STDP differ greatly between large and small spines, supporting the idea that they might store different traces of memory (Matsuzaki et al., 2004). However, it remains a challenging task to find experimental settings that will allow us to test whether such spine morphologies indeed affect memory formation *in vivo*.

## Coexistence of Synaptic Plasticity Activated by High and Low Repeat Number STDP Protocols

Neuromodulatory transmitters and spine morphology are not the sole microenvironmental parameters that decide whether a spine can undergo t-LTP. Rather, the pattern of bAPs and the number of STDP paradigm repeats constitute an electrical code that can recruit a given synaptic spine for t-LTP while neglecting others. Timing-dependent LTP can be induced reliably throughout the brain using roughly 50–300 repeats of 1:1 pairings at low frequency (<2 Hz; see e.g., Couey et al., 2007; Seol et al., 2007; Campanac and Debanne, 2008; Edelmann and Lessmann, 2011; Feldman, 2012; Banerjee et al., 2014; Huang et al., 2014; Yang and Dani, 2014; Cui et al., 2015; Edelmann et al., 2015; Mishra et al., 2016; Tigaret et al., 2016). Robust t-LTP was also reported by higher pairing frequency up to 5 Hz (Wittenberg and Wang, 2006; Carlisle et al., 2008; Tigaret et al., 2016), which has been described to reduce the number of repeats that is required to successfully induce t-LTP (Pike et al., 1999; Hardie and Spruston, 2009). To warrant physiological relevance of STDP experiments it seems reasonable to minimize the number of repeated pre- and postsynaptic stimulations for induction of plasticity in electrophysiological experiments. Although such a physiological t-LTP approach would potentially help to identify the minimum requirements to encode a memory trace with a set of synapses, low repeat and low frequency STDP paradigms have only sparsely been investigated. Froemke et al. (2006) demonstrated t-LTP in layer 2/3 pyramidal neurons by using a 1:1 STDP paradigm with few repeats (<20 at 0.2 Hz). Furthermore, they demonstrated an increase in t-LTP magnitude with increasing number of repeats, which was saturated by 60 repeats (Froemke et al., 2006). Likewise, for dissociated cultures of hippocampal neurons at 9–15 days *in vitro*, Zhang et al. (2009) reported t-LTP that was induced by just 20 repeats of a 1:1 STDP paradigm. The threshold number of pre- and postsynaptic spikes required to induce t-LTP in these hippocampal cultures was shown to decrease upon exogenous application of DA (Zhang et al., 2009). A comparably low number of pre/post spike pairings was also described to be sufficient for inducing t-LTP in medium spiny neurons in acute striatal slices. The authors observed successful t-LTP by 5–15 pairings of coincident pre- and postsynaptic spikes. This form of plasticity relied on activation of presynaptic type-1 cannabinoid receptors and activation of postsynaptic transient receptor potential (TRP) vanilloid type-1 Ca^2+^ channels. However, this type of t-LTP was induced by post-pre pairings at 1 Hz (i.e., anti-hebbian synaptic plasticity; Cui et al., 2015, 2016). In our lab, we managed to induce low repeat t-LTP in CA1 pyramidal neurons in acute hippocampal slices. In slices from rats we observed significant potentiation in response to just 12 repeats (at 0.5 Hz) of a 1:1 paradigm while even just six repeats were efficient for t-LTP induction in CA1 in mice (Cepeda-Prado et al., unpublished data). However, our preliminary results suggest that the activated signaling cascades that allow expression of t-LTP under these very physiological conditions are quite different from the respective results obtained with higher repeat numbers. Thus, neuromodulation by DA and BDNF, pre- vs. postsynaptic sites of t-LTP expression, and the required sources for Ca^2+^ elevation to trigger t-LTP seem to be substantially different. With respect to BDNF contribution to t-LTP, we could show recently in CA1 neurons in acute hippocampal slices that an autocrine signaling loop consisting of postsynaptic secretion of endogenous BDNF, followed by postsynaptic TrkB activation, and subsequent AMPAR insertion mediates 1:4 induced t-LTP (25–30 repeats; Edelmann et al., 2015). In dissociated cultures of hippocampal neurons, Lu et al. (2014) observed presumably postsynaptic secretion of overexpressed BDNF in response to repeated 1:1 pairings. With 40 repeats they reported a significant level of secretion which steadily increased up to STDP protocols employing 160 repeats (Lu et al., 2014). Whether these data can be taken to indicate that low numbers of repeats of STDP paradigms are insufficient to secrete BDNF from postsynaptic structures remains to be investigated. Clearly, further studies employing e.g., Ca^2+^ imaging in synaptic spines are critically needed to determine whether high and low repeat STDP paradigms in fact recruit distinct synapses of a neuron.

It would be extremely interesting to find out whether the cellular findings in acute slices *in vitro* that we discuss here can be transferred to processes of memory formation in behaving animals. At present, such an approach is to our knowledge not available. However, computational modeling of electrical signals in CA1 circuits on the basis of synaptic properties that are discussed in this article might set up a framework to disentangle how cellular mechanisms of t-LTP and t-LTD can generate memory relevant changes e.g., in electrical oscillations generated in a population of CA1 pyramidal neurons. This modeling could yield new hypotheses how altered properties of CA1 network oscillations might reflect memory formation in behaving animals that could be tested using electrophysiological *in vivo* recordings.

## Parallel Forms of Synaptic Plasticity by Recruiting Heterosynaptic Plasticity

So far we focused here on the coexistence of different types of synaptic plasticity, employing different signaling mechanisms at the same subset of synapses, and at different locations (including spines) along CA1 dendrites that might be susceptible to undergo t-LTP in response to divergent STDP protocols (associative or input-specific synaptic plasticity; compare e.g., Buonomano and Merzenich, 1996; Verhoog et al., 2013). However, hetero-synaptic plasticity would be helpful to enhance the storage and processing capacity of brain circuits, because information can be encoded not only in the input-specific path, but also in the heterosynaptically strengthened input independent path. In addition, depending on the type of heterosynaptic plasticity that is established, it will also prevent saturated excitation of circuits and thus keep the brain functional for processing information. To this aim, LTP is often accompanied by heterosynaptic LTD, and vice versa (see e.g., Yu and Goda, 2009; Chistiakova et al., 2014; Fernandes and Carvalho, 2016). Non-associative (= input independent) processes and hebbian (= input specific) synaptic plasticity can act in concert (Lynch et al., 1977; Han and Heinemann, 2013). These coexisting processes—although often neglected—have been described also for STDP (Kodangattil et al., 2013; Chistiakova et al., 2015; Jedlicka et al., 2015). In terms of neuromodulatory functions in STDP, BDNF as well as DA can be secreted by associative plasticity and transiently or persistently increase synaptic transmission at non-stimulated nearby synapses of the same neuron (compare Figure 1). Such mechanisms might thereby serve in forming additional memory traces at nearby synapses.

Taken together these additional heterosynaptic effects of synaptic plasticity that are induced by STDP paradigms might lead to even more and sophisticated ways to strengthen or weaken synapse function as cellular correlate for learning and memory.

## Summary

Summarizing and collecting evidence of published and our own unpublished data, we think that synaptic plasticity at SC CA1 synapses has multiple facets and mechanisms. The magnitude of t-LTP and the locus of t-LTP expression depend on dendrite subtype, spine structure and location, location of synapses at proximal vs. distal dendrites, effective neuromodulation and background brain activity. Furthermore, we believe that the different mechanisms for encoding or processing memory can coexist simultaneous and independently at the same subset of synapses. However, so far we have just begun to understand how these processes interact at the cellular level and can only speculate how they might be involved in learning and memory *in vivo*.

Clearly, a plethora of new experiments will be required to translate how the *in vitro* data reviewed in this article might contribute to memory formation *in vivo*.

## Author Contributions

EE and VL designed the outline of the article. EE, EC-P and VL wrote the text. EE and VL prepared the figures.

## Conflict of Interest Statement

The authors declare that the research was conducted in the absence of any commercial or financial relationships that could be construed as a potential conflict of interest.
